# Early Zinc Supplementation and Enhanced Growth of the Low-Birth Weight Neonate

**DOI:** 10.3889/oamjms.2015.007

**Published:** 2014-12-17

**Authors:** Ola El-Farghali, Mohamed Abd El-Wahed, Nayera E. Hassan, Safaa Imam, Khadija Alian

**Affiliations:** 1 *Ain Shams University, Children’s Hospital Cairo, Abbassia sq., Cairo 11351, Egypt*; 2 *National Research Centre - Biological Anthropology, National Research Centre, ElBuhose street, Dokki, Giza, Egypt*

**Keywords:** low birth weight, zinc, supplementation, catch-up growth, micronutrients

## Abstract

**BACKGROUND:**

Nutritional deficits are almost universal in Low-Birth Weight babies. Zinc is essential for normal infant growth and its supplementation assists growth probably through insulin-like growth factor-1.

**AIM:**

This double-blind randomized-controlled trial aimed at evaluating the role of zinc in catch-up growth of low-birth-weight infants and investigating its proposed mediator.

**MATERIAL AND METHODS:**

The study was conducted in Ain Shams University Maternity Hospital. Two hundred low-birth-weight neonates were simply randomized to either oral zinc therapy or placebo. Anthropometric measurements were recorded at birth, 3, 6, and 12 months; including weight, recumbent length, head, waist, chest, and mid-upper arm circumferences, and triceps and sub-scapular skin fold thickness.

**RESULTS:**

We found that initial and 3-months measurements, except weight, were comparable in the 2 groups. All measurements at 6- and 12-months, except sub-scapular skin-fold-thickness, were significantly higher in zinc group than placebo. Catch-up growth, at 12-months, was significant in zinc group and was significantly higher in appropriate-for-gestational-age vs. small-for-gestational-age, in preterm vs. term, and in male vs. female infants. The median 6-months insulin-like growth factor-1 levels were significantly higher in zinc group.

**CONCLUSION:**

We conclude that early start of oral zinc supplementation in low-birth-weight neonates assists catch-up growth, probably through rise of insulin-like growth factor-1.

## Introduction

Low birth weight (LBW) has been defined by the World Health Organization (WHO) as weight at birth less than 2,500 grams, irrespective to gestational age. Its prevalence is higher in developing than developed countries [1]. This condition can be due to premature birth and/or intrauterine growth restriction [2].

Nutritional deficits, including micronutrients, are almost universal in LBW babies; making them a main target for early intervention [3]. Being an integral part of more than 100 enzyme systems in human body, zinc is essential for normal infant growth and development and its deficiency contributes greatly to impaired growth [4]. This growth-enhancing action is strongly assumed to be mediated by insulin-like growth factor (IGF)-1 [5].

So, the aim of this trial was to evaluate the growth promoting effect of zinc supplementation in LBW neonates, and to clarify its relation to the main growth hormone mediator, IGF-1.

## Patients and Methods

This double-blind randomized placebo-controlled trial; neither the main investigators nor the neonates’ parents knew whether the subject was receiving drug or placebo, was conducted over the period from October 2010 through May, 2012. All LBW neonates, whether term or preterm, appropriate for gestational age (AGA) or small for gestational age (SGA), were eligible. Exclusion criteria included: delayed establishment of enteral feedings, birth weight <1500 g, gestational age <28 weeks, congenital malformations, chromosomal aberrations or evidence of considerable illness. The study was approved by the Local Ethical and Research Committee; registered approval number is 09202. Written consents were taken from parents.

The study comprised 200 LBW neonates whom were randomized; by simple randomization, into two groups: Zinc group; including neonates who received oral zinc therapy at a dose of 10 mg/day, and Placebo group. Randomization tables were kept with an independent investigator and were only revealed at the time of final statistical analysis. The drug/placebo containers were obtained, labeled and numbered by the same independent investigator.

### Oral therapy (drug or placebo)

Bottles used for oral therapy, either zinc sulfate or placebo, were of the same shape and size. The concentration of oral zinc solution was 2 % (20 mg/ml). To supplement 10mg/day, each infant received ½ ml/day. Equal volumes of placebo (distilled water) were used for placebo group. The bottles were numbered serially by the independent investigator and the codes were kept confidential until the study was finished. Therapy started with the start of oral feeds and lasted for 6 months.

Anthropometric measurements were recorded at birth and then 3-monthly, including weight, length, head circumference, waist, chest and mid-upper arm circumferences, and triceps and sub-scapular skin fold thickness. All measurements were taken on the right side and were based on the Anthropometry Procedures Manual provided by the US Center for Disease Control (CDC) [6].

### Laboratory Methods

Venous blood samples were withdrawn from all recruited neonates: initially – within first 3 days after birth – for baseline serum zinc measurement, and at the 6^th^ months for IGF-1 measurement. Samples were centrifuged and sera were stored at -70°C until analysis. Serum zinc was measured using Quimica Ckininca Aplicada S.A kit for colorimetric in vitro zinc measurement. Serum IGF-1 was measured using DIA (IGF1-EASIA).

Statistical analysis was performed using Statistical Package for Social Sciences (SPSS) version 16.0. Data for the two independent groups were compared using independent student’s t-test and the mean difference [MD] with its 95% confidence interval [95% CI] (for parametric variables), Mann-Whitney’s U-test (for non-parametric variables) and chi-squared test as well as the relative risk [RR] and its 95% CI (for categorical variables). The number needed to treat (NNT) was calculated as the reciprocal for the absolute risk reduction (ARR). The cutoff values for the tenth percentiles of weight, height and HC were set according to the CDC charts for growth of infants. The significance level was set at 0.05.

## Results

Two hundred neonates were recruited; 121 (60.5%) males and 79 (39.5%) females, 48 (24%) term and 152 (76%) preterm, with a mean gestational age of 35.08 ± 1.93 (32 – 40) weeks and a mean birth weight of 1966.29 ± 307.61 (1500 – 2490) grams. One hundred and twenty nine (64.5%) neonates were appropriate for gestational age (AGA), while 71 (35.5%) were small for gestational age (SGA).

The median initial serum zinc concentration in studied neonates was 111.35 μg/dl (31 – 554 μg/dl; IQR 83.3 – 145.5 μg/dl).

Zinc group included 108 neonates and Placebo group included 92 neonates. The study course and overall drop-out rate [29/200 (14.5%)] are presented in a flow diagram.

**Figure 1:**
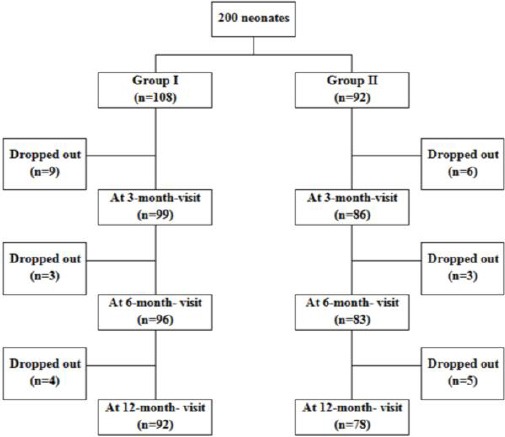
*Flow-Diagram showing Study Course*.

Neonates of both groups were non-significantly different regarding gender distribution (62/108 males and 46/108 females in Zinc group vs. 59/92 males and 33/92 females in Placebo group; p = 0.332), gestational age (34.95 ± 1.79 vs. 35.28 ± 2.13 wk, respectively; p = 0.237), birth weight (1944.63 ± 307.25 vs. 1991.73 ± 307.76 gm, respectively; p = 0.282), and distribution of AGA and SGA cases (69/108 AGA and 39/108 SGA vs. 60/92 AGA and 32/92 SGA, respectively; p = 0.845).

Initial anthropometric measurements were also non-significantly different between the 2 groups (p >0.05 for all parameters).

Median (IQR) initial serum zinc levels were non-significantly different between the 2 groups [114.05 (87.15–144.95) vs. 107.15 (73.38–154.4), respectively; p = 0.225].

At 3-month-visit, weight was the only measurement that significantly increased in Zinc group compared to Placebo group with mean values of 3964.14 ± 947.23 vs. 3665.69 ± 888.63 gm, respectively (p = 0.029; MD = 298.44, 95% CI = 30.7 to 566.2).

**Figure 2:**
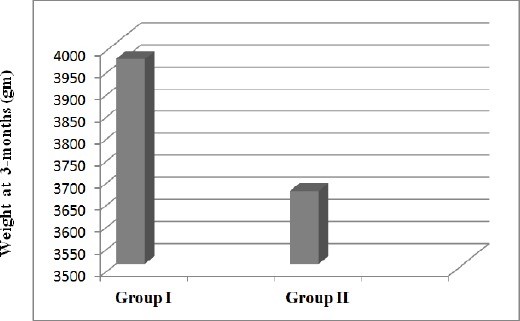
*Stacked column showing significantly higher weight in zinc than placebo neonates at 3-month-visit*.

The percentage of SGA infants who had their weight values above 10^th^ percentile for age was not statistically different between the 2 groups (10 vs. 8%, respectively; p = 0.6, RR = 1.24, 95% CI = 0.49 to 3.12, NNT = 51).

**Table 1:** Comparison of zinc and placebo neonates regarding 6-months anthropometric measurements.

	Group I [Zinc Group] (n=96)	Group II [Placebo Group] (n=83)	P*	MD (95% CI)
**Weight (9m) Min-Max Mean ± SD**	4800 – 8800 6867.71 ± 1247.4	4300 – 8800 6350.6 ± 1134.9	0.006 S	427.27 (123.44 to 731.11)

**Length (cm) Min-Max Mean ± SD**	51.5 – 73 63.14 ± 3.93	52.5 – 72.5 61.93 ± 3.49	0.033 S	1.2 (0.1 to 2.3)

**HC (cm) Min-Max Mean ± SD**	36.3 – 45.8 41.16 ± 2.17	34 – 47 40.35 ± 2.16	0.013 S	0.81 (0.17 to 1.45)

**Waist Circumference (cm) Min-Max Mean ± SD**	31.5 – 45 38.61 ± 3.11	32 – 43.5 37.73 ± 2.43	0.04 S	0.87 (0.04 to 1.7)

**Chest Circumference (cm) Min-Max Mean ± SD**	35 – 48.5 41.54 ± 3.04	32.5 – 46 40.59 ± 2.74	0.031 S	0.95 (0.09 to 1.8)

**MAC (cm) Min-Max Mean ± SD**	8.9 – 17.4 13.71 ± 1.84	9 – 17 13.07 ± 1.71	0.018 S	0.64 (0.11 to 1.16)

**Triceps SFT (mm) Min-Max Mean ± SD**	6 – 11 9.79 ± 1.41	5 – 11 9.29 ± 1.49	0.025 S	0.49 (0.06 to 0.92)

**Subscapular SFT (mm) Min-Max Mean ± SD**	4.5 – 8.5 8.12 ± 0.74	4.5 – 8.5 8 ± 0.86	0.316 NS	0.12 (-0.12 to 0.35)

* Analysis using Independent Student’s t-Test. MAC= Mid-arm circumference, SFT= Skin fold thickness; MD (95% CI) mean difference and its 95% confidence interval; S= significant, HS= highly significant, NS= non-significant.

At 6-month-visit, all anthropometric measurements, except subscapular skin fold thickness, were significantly higher in Zinc group compared to Placebo group.

**Figure 3:**
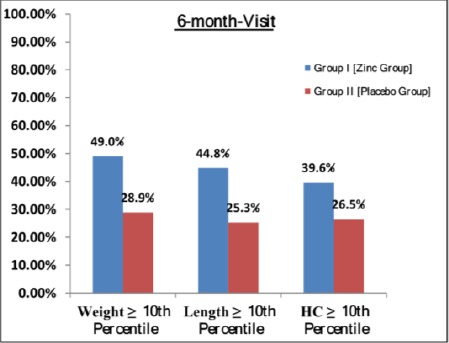
*Bar chart showing that percentage of infants above 10^th^ percentile for weight and length was significantly higher in zinc than placebo groups at 6-months, but not for head circumference*.

At that time, the proportions of SGA infants who showed catch-up growth (i.e. had their weight and length values above 10^th^ percentile for age) were significantly higher in Zinc than Placebo groups; for weight (49 vs. 29%, respectively; p = 0.006, RR = 1.69, 95% CI = 1.14 to 2.51, NNT = 5) and for length (45 vs. 25%, respectively; p = 0.007, RR = 1.77, 95% CI= 1.15 to 2.72, NNT = 5). For head circumference, higher percentage of SGA infants passed the 10^th^ percentile in Zinc than Placebo groups, but this was statistically non-significant (40 vs. 27%, respectively; p>0.05).

The median IGF-1 levels measured at 6 months were significantly higher in Zinc compared to Placebo groups [median (IQR): 90 (51.7–113.8) vs. 74 (46–101) ng/ml), respectively; p = 0.023]. However, IGF-1 levels did not show significant correlation with any of the anthropometric measurements in the two groups [r = 0.13, p = 0.2 for weight, r = 0.02, p = 0.8 for length, and r = 0.03, p = 0.7 for head circumference, in Zinc group].

At 12-month-visit, again, all anthropometric measurements, except subscapular skin fold thickness, were significantly higher in Zinc group.

**Table 2:** Comparison of zinc and placebo neonates regarding 12-months anthropometric measurements.

	Group I [Zinc Group] (n=92)	Group II [Placebo Group] (n=78)	P*	MD (95% CI)
**Weight (gm) Min-Max Mean ± SD**	5650 – 11650 8888.9 ± 1369.5	4900 – 11650 8134.8 ± 1377.9	<0.001 HS	752.2 (336.3 to 1168.03)

**Length (cm) Min-Max Mean ± SD**	61.9 – 81.9 73.17 ± 4.12	61 – 80 70.46 ± 3.7	<0.001 HS	2.7 (1.5 to 3.9)

**HC (cm) Min-Max Mean ± SD**	38.5 – 49.5 44.92 ± 2.49	35 – 52 44.63 ± 2.71	0.001 S	1.3 (0.49 to 2.07)

**Waist Circumference (cm) Min-Max Mean ± SD**	32 – 51 41.74 ± 3.32	35 – 48.5 40.78 ± 2.85	0.046 S	0.96 (0.02 to 1.9)

**Chest Circumference (cm) Min-Max Mean ± SD**	35.5 – 51.5 44.13 ± 3.37	39 – 50.5 42.83 ± 3.99	0.022 S	1.31 (0.19 to 2.42)

**MAC (cm) Range Mean ± SD**	11.5 – 21 16.18 ± 2.21	11 – 20.5 15.27 ± 1.94	0.005 S	0.91 (0.27 to 1.54)

**Triceps SFT (mm) Min-Max Mean ± SD**	9.5 – 13 12.78 ± 0.59	9 – 13 12.46 ± 0.94	0.006 S	0.33 (0.09 to 0.56)

**Subscapular SFT (mm) Min-Max Mean ± SD**	7 – 9 8.95 ± 0.26	7.5 – 9 8.96 ± 0.21	0.881 NS	-0.01 (-0.08 to 0.07)

* Analysis using Independent Student’s t-Test. MAC= Mid-arm circumference, SFT= Skin fold thickness; MD (95% CI)= mean difference and its 95% confidence interval; S= significant, HS= highly significant, NS= non-significant.

**Figure 4:**
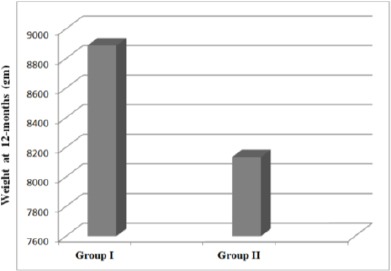
*Stacked column showing significantly higher weight in zinc than placebo neonates at 12-month-visit*.

At that time, the proportions of SGA infants who had their head circumference – as well as weight and length – values above 10^th^ percentile for age were significantly higher in Zinc group; for head circumference (58 vs. 39%, respectively; p = 0.011, RR = 1.5, 95% CI = 1.08 to 2.07, NNT = 5), for weight (56 vs. 31%, respectively; p = 0.001, RR = 1.79, 95% CI = 1.23 to 2.59, NNT = 4) and for length (65 vs. 39%, respectively; p = 0.001, RR = 1.77, 95% CI = 1.22 to 2.27, NNT = 4).

**Figure 5:**
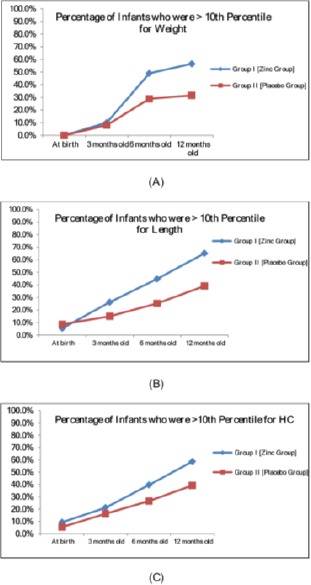
*Curve plot showing that catch-up of weight was obvious in group I (Zinc Group) infants compared to group II (Placebo Group) from 6^th^ month (A), catch-up of length was even earlier at the 3^rd^ month (B), and catch-up for HC was only observed by the 12^th^ month (C)*.

Upon comparing SGA to AGA infants – within Zinc group – as regards increment of the 3 basic anthropometric measurements at 12-month-visit, length was the only parameter that significantly increased in SGA infants (72.25 ± 4.69 vs. 69.89 ± 3.65 cm, respectively; p = 0.033, MD = 2.4, 95% CI = 0.19 to 4.5).

In the same regard, length was the only parameter that showed catch-up in a significantly larger proportion of SGA infants out of Zinc group compared to Placebo (57 vs. 27%, respectively; p = 0.017, RR = 2.09, 95% CI = 1.08 to 4.03, NNT = 3).

At 12-month-visit, preterm infants of Zinc group showed significant increase of weight (8840.6 ± 1391.3 vs. 8016.4 ± 1399.6 gm, respectively; p = 0.013, MD = 824.2, 95% CI = 325.1 to 1323.4), highly significant increase in length (72.8 ± 4.1 vs. 70.1 ± 3.6 cm, respectively; p < 0.001, MD = 2.7, 95% CI = 1.3 to 4.1), and significant increase in head circumference (44.5 ± 2.5 vs. 43.2 ± 2.7 cm, respectively; p = 0.006, MD = 1.3, 95% CI = 0.4 to 2.2).

Zinc-supplemented term infants, however, showed improved length and head circumference values compared to placebo infants (p = 0.013 and 0.04, respectively), but both groups were comparable for weight increment (p = 0.114).

Male infants of Zinc group had their weight, length and HC values, at the 12-month-visit, significantly higher than those of Placebo group (p < 0.001 for weight and length, p = 0.002 for HC). On the other hand, female infants were comparable between the 2 groups regarding all measurements (p > 0.05).

Importantly, none of our Zinc-supplemented infants suffered any of the known side effects of zinc such as vomiting, diarrhea, abdominal cramps, or loss of appetite.

## Discussion

Many studies concerned with the effects of Zinc supplementation on growth during fetal and early postnatal life were done. Our study focused on the growth promoting effect of high-dose zinc supplementation over a six months period, starting from day 1 of enteral feeding in LBW neonates, in comparison to placebo. Initial serum zinc levels measured in our patients were almost similar between the 2 groups. This supported the hypothesis that effects of zinc supplementation on growth, if any, are clearly dependant on the dose regimen not on body reserve.

The adequate intake (AI) for zinc is 2mg/day for both males and females [7], while its recommended daily allowance (RDA) in 0-6 months infants is 5-10 mg/day [8].

The side effects of zinc overdose are very rare and none were reported with doses <25 mg/day [8] and they include vomiting, diarrhea, abdominal cramps, loss of appetite, and manifestations of copper and iron deficiency [9]. None of our babies developed any of these known side effects. In addition, the same dose as ours was used for one week in neonates with idiopathic hyperbilirubinemia in an Indian study by Kumar et al. (2014), and they reported no side effects [10].

Many studies and systematic reviews of trials have reported improved weight gain and linear growth in LBW infants supplemented with Zinc in different dosage regimens and for different durations [5, 11-13]. Regarding head circumference, significant increment was also shown following 6-months-period of 5mg/day supplementation [14]. Even when supplemented to 4-17 months old infants, zinc conferred a beneficial enhancement of head growth [15].

So, it could be now considered as a fact that preventive zinc supplementation in populations at risk of zinc deficiency increases linear growth and weight gain among infants and young children [16].

On the other hand, a double-blind placebo-controlled trial done on 3 to 9 months old infants with non-organic failure to thrive showed no growth promoting effect of zinc being supplemented for 12 weeks [17]. This could be explained by other nutrient deficiencies which can interfere with the growth promoting effect of zinc [18]. In another study, the research workers supplemented 128 SGA infants with oral zinc for 6 months and reported no effect on their growth [19]; this could be explained by their use of low doses of zinc. Moreover, supplementation of low doses, such as 3 mg/day did not improve growth despite increasing serum zinc levels in SGA infants [20].

The finding of better length increment in SGA vs. AGA infants was also reported in a Chilean study [21] and our finding of their poorer weight gain was, again, similar to some earlier studies [22]. This result is also supported by the well-recognized preferential action of zinc on cartilage and bone growth [23, 24] and by the report of a recent meta-analysis of 24 studies concluding that benefits from zinc supplementation are mainly on linear growth [25].

Rapid weight gain in infants is associated with higher circulating IGF-1 [26]. The relation between IGF-1 and zinc was suggested many years ago by Ninh et al. (1996) and recently by Alves (2012) who reported significantly increased IGF-1 plasma levels after 3 months of 5mg daily supplement of zinc in 6-9 years old children [18, 27].

In an Egyptian study, significant elevation of IGF-1 correlated with elevation of serum zinc levels and with increased height of 50 pre-pubertal short children following 3 months of oral zinc supplementation [28]. Adding to the same fact, some studies reported decreased IGF-1 levels in zinc deficient patients [29, 30].

While our zinc-supplemented males, rather than females, had significant growth enhancement, some studies reported better linear growth in female more than male LBW infants [11, 12].

In summary, the present results support the reports of positive growth promoting effect induced by zinc supplementation in LBW neonates and its association with elevated IGF-1 which is strongly suggested to mediate the catch-up growth induced by zinc. This study is unique in the earliest supplementation of the highest RDA for zinc.
